# Hypersensitivity to parenteral antibiotics in patients with cystic fibrosis

**DOI:** 10.1186/2045-7022-4-S3-P66

**Published:** 2014-07-18

**Authors:** Jobst Roehmel, Carsten Schwarz, Anne Mehl, Philippe Stock, Doris Staab

**Affiliations:** 1 Charité Universitätsmedizin Berlin, Pediatrics, Division of Pneumonology & Immunology, Germany

## Background

Hypersensitivity reactions to parenteral administered antibiotics (HRPA) are a substantial problem in managing pulmonary disease in Cystic Fibrosis (CF), especially in advanced CF. This group of patients requires a life long antibiotic treatment with extremely high cumulative doses compared to other patients. In our daily routine we observed a growing number of hypersensitivities. Therefore we conducted this observational study to assess HRPA´s impact on the daily clinical work with CF, as well as it's nature, frequency and predisposing risk factors.

## Methods

By reviewing medical records and conducting interviews, age, sex, FEV1, ΔF508-genotype, onset and duration of pseudomonal colonisation, allergy history (including IgE serum levels, past ABPA and results of screening tests for inhalative aeroallergens), parenteral antibiotic exposure and HRPA (timing, symptoms and treatment) were recorded. Included were all paediatric and adult patients at our centre with >3 intravenous antibiotic treatment courses.

## Results

Of 100 patients included in the study, 60 had ≥1 HRPA. Overall, 3205 antibiotic courses with 185 HRPA were ascertained. 15% of HRPA met the criteria for anaphylaxis. Symptoms were mostly dermal (53%). 81% of all and 80% of anaphylactic HRPA occurred during days 1-4. Approximately 10% of all treatment courses with cefepime and piperacillin/tazobactam caused HRPA. The number of years with pseudomonal colonisation and the cumulative annual exposure of the given antibiotic were significant risk factors for HRPA in our patient cohort.

## Conclusions

Our results demonstrate that HRPA with a prevalence of 60% are very relevant. During days 1-4 of antibiotic treatment courses patients might be at elevated risk to experience HRPA. HRPA appear to be drug-specific and to be dependent on cumulative annual drug exposure of the given drug. High cumulative dose of the same antibiotic over a short period of time may lead to a higher risk of HRPA than the same dose over a longer period of time. Is this an argument to change the therapeutic regimen more often? Diagnostic algorithms for this CF-specific problem should be developed further. Besides recent publications about drug specific lymphocytes in patients with CF, we believe that further elucidation of HRPA´s immunological mechanisms, also concerning immediate reactions and diagnostics, is needed.

